# Supplementary material for: Dynamic activation catalysts for CO2 hydrogenation
Source: Nat Commun. 2025 Oct 22;16:9349. doi: 10.1038/s41467-025-64417-8 (PMC12546630; doi:10.1038/s41467-025-64417-8)
Supplement: Supplementary file 1 — Supplementary Information [file 41467_2025_64417_MOESM1_ESM.pdf]

Supplementary Information

Dynamic Activation Catalysts for CO_2 Hydrogenation

Zhewei Zhang[1], Jun Yao[1], Chenyang Shen[1], Fengfeng Li, Changshun Deng, Taotao Zhao, Xuefeng Guo, Yan Zhu, Xiangke Guo, Nianhua Xue, Luming Peng, Weiping Ding*

Key Lab of Mesoscopic Chemistry, School of Chemistry and Chemical Engineering, Nanjing University, Nanjing 210023, China

[1]These authors contributed equally.

* Correspondance author. Email: Dingwp@nju.edu.cn

Supplementary Figures, Tables and Calculation Details

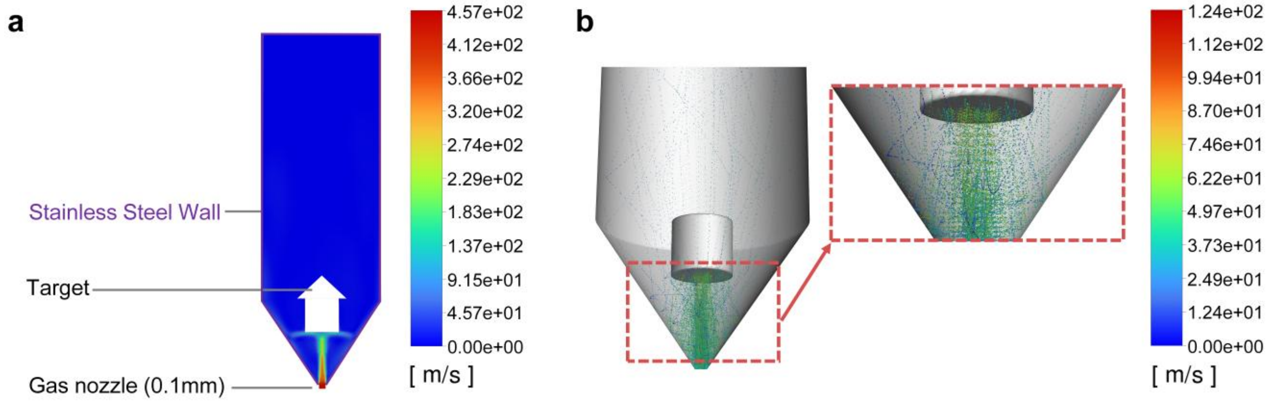

Supplementary Figure 1. Simulations of the fluid dynamics in the dynamic activation reactor. (a) The 2D section of the velocity distribution of the gas flow; (b) Instantaneous snapshots of catalyst particles carried by the gas flow and the size of catalyst particles corresponding to randomly generated particles of different sizes. (P: 2.0 MPa; T: 300 °C; $3H_2/CO_2$; Gas line velocity at the inlet: ~452m/s, Velocity of catalyst particles at impact with the target: ~75m/s)

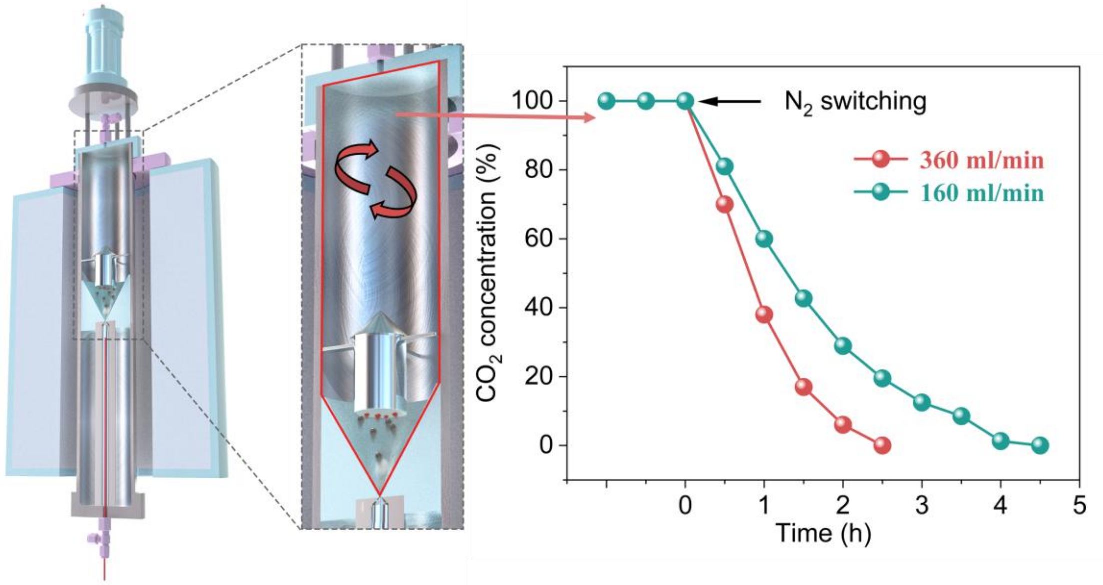

Supplementary Figure 2. Test of transient time of complete replacement of CO_2 in dynamic activation reactor by N_2. (P: 2.0 MPa; T: 300 °C; Flow rate: 160, 360 ml/min)

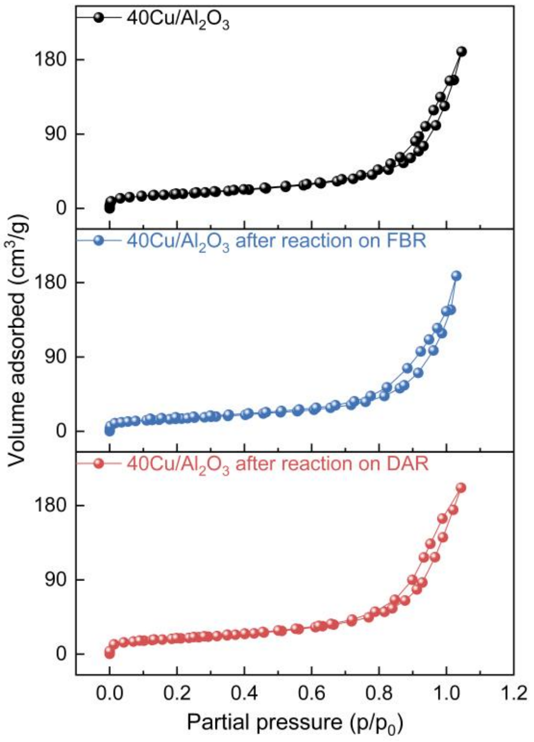

Supplementary Figure 3. N_2 sorption isotherms of the samples. (DAR: dynamic activation reactor, FBR: traditional fixed bed reactor)

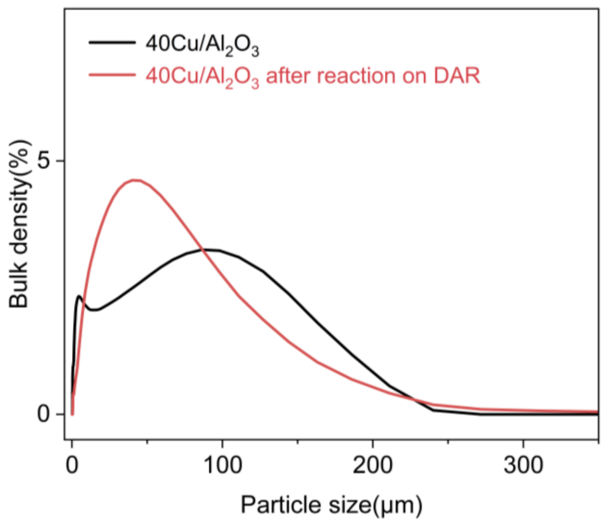

Supplementary Figure 4. Catalyst particle size distribution measured using laser scattering particle size analyzer before and after DAR reaction.

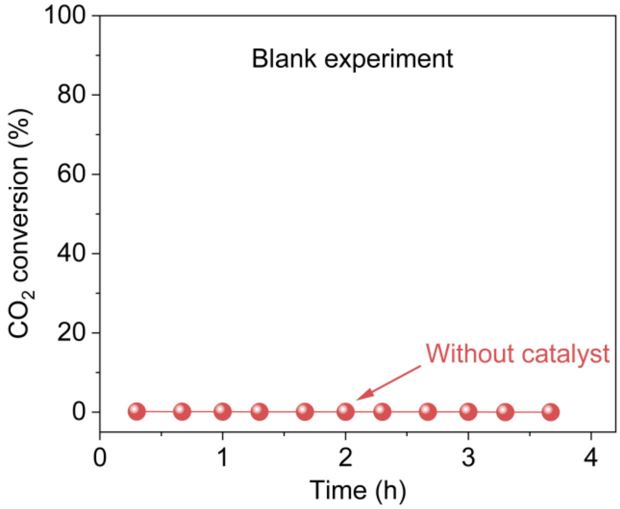

Supplementary Figure 5. Blank test of CO_2 hydrogenation measured using the dynamic activation reactor (DAR). (P: 2.0 MPa; T: 300 °C; H_2: CO_2: Ar = 69: 23: 8; Flow rate: 360 ml/min)

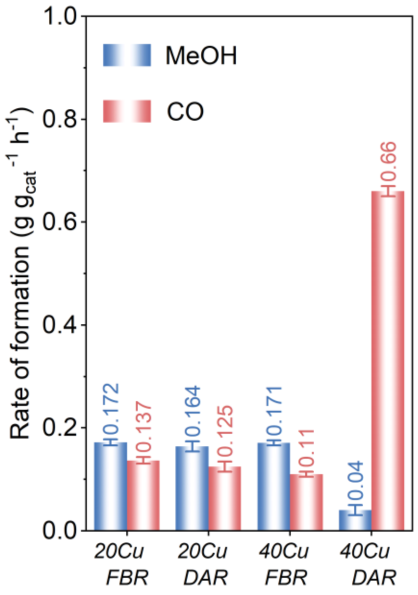

Supplementary Figure 6. Formation rates of CO and methanol over 20Cu/Al_2O_3 and 40Cu/Al_2O_3 measured in dynamic activation reactor (DAR) (P: 2.0 MPa; T: 300 °C; Cat: 1 g; Flow rate: 360 ml/min) and fixed bed reactor (FBR) (P: 2.0 MPa; T: 300 °C; Cat: 0.2 g; Flow rate: 72 ml/min). The error bars represent the standard deviation from three independent measurements.

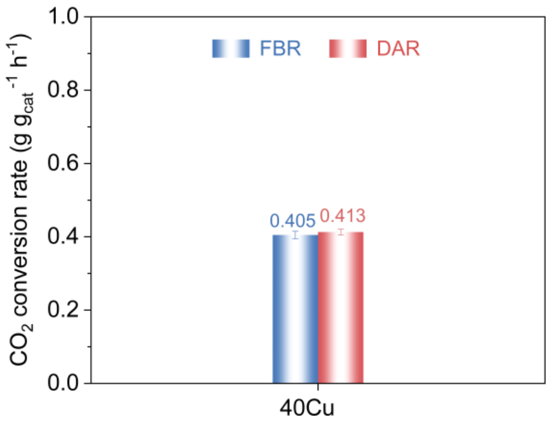

Supplementary Figure 7. CO_2 conversion rate over 40% Cu/Al_2O_3 measured under dynamic activation (Cat: 1g; Flow rate: 160 ml/min; GHSV: 9600 mLg_{cat}^{-1} h^{-1}) and traditional fixed bed reactor (FBR) (Cat: 0.2g, Flow rate: 32 ml/min; GHSV: 9600 mLg_{cat}^{-1} h^{-1}). (P: 2.0 MPa; T: 300 °C; H_2: CO_2: Ar = 69: 23: 8). The error bars represent the standard deviation from three independent measurements.

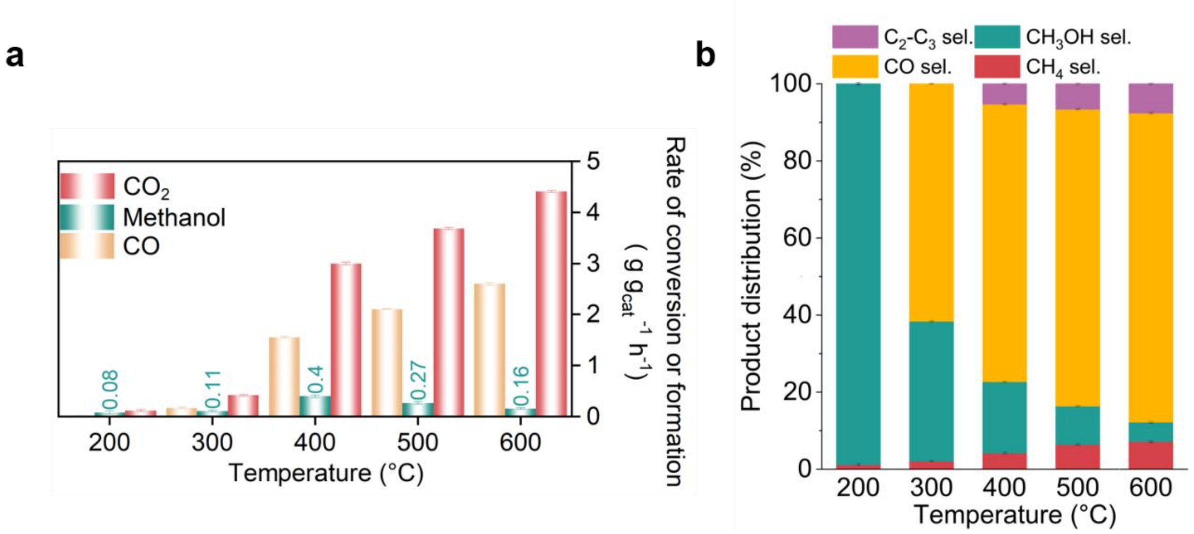

Supplementary Figure 8. (a), (b) Catalytic performance of 40Cu under different temperature conditions in fixed bed reactor (FBR). (P: 2.0 MPa; Cat: 0.2 g; Flow rate: 72 ml/min). The error bars represent the standard deviation from three independent measurements.

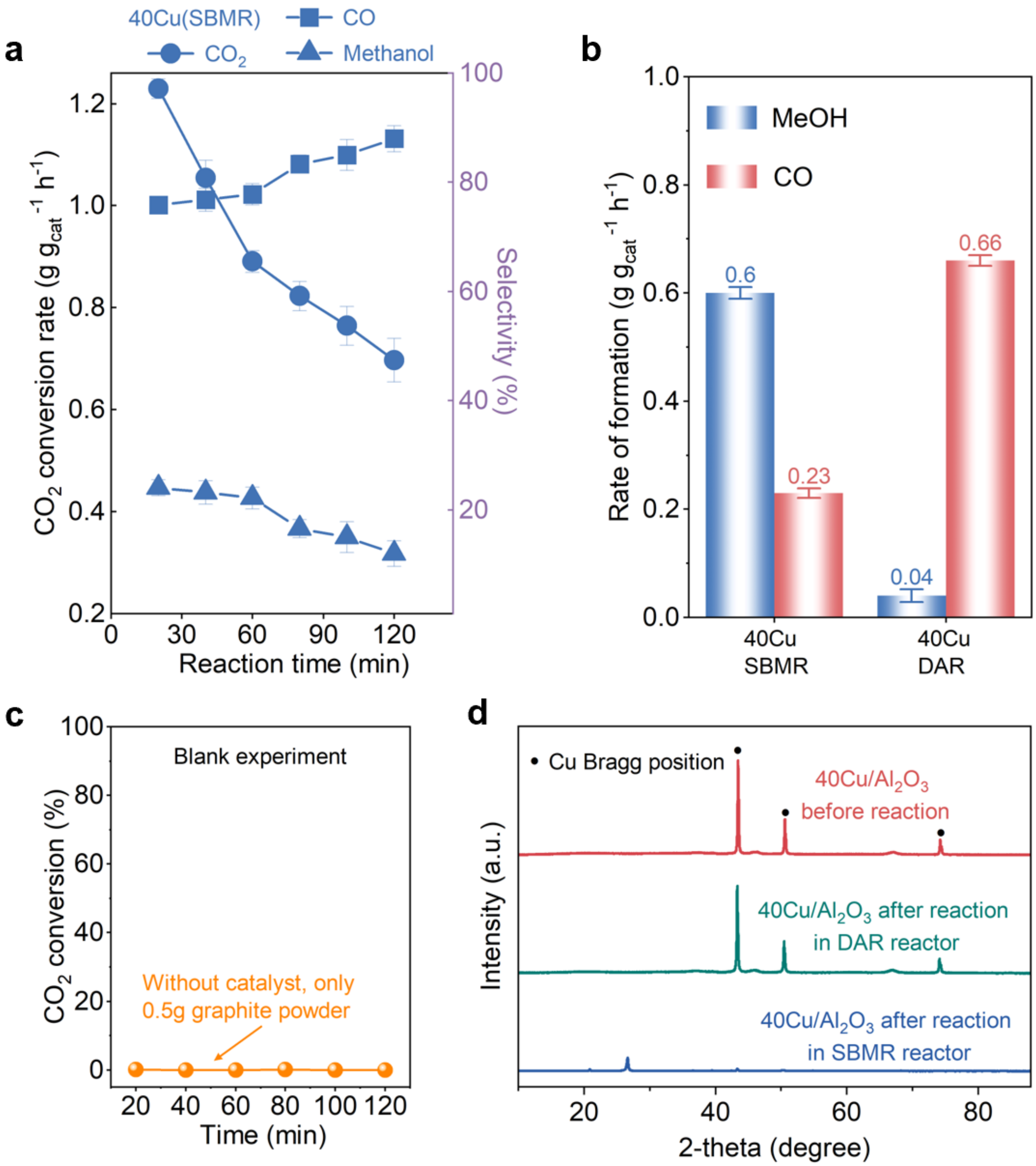

Supplementary Figure 9. (a) Catalytic performances of 40Cu in stirred ball mill reactor (SBMR). (b) Formation rates of CO and methanol over 40Cu measured in stirred ball mill reactor (SBMR) and dynamic activation reactor (DAR). (c) Blank test of CO2 hydrogenation measured using the stirred ball mill reactor (SBMR). (d) Rietveld refinement of XRD patterns of catalyst 40Cu. (P: 2.0 MPa; T: 300 °C; Cat: 0.2 g; lubricants (graphite powder): 0.5 g; Flow rate: 72 ml/min, if unspecified). The error bars represent the standard deviation from three independent measurements.

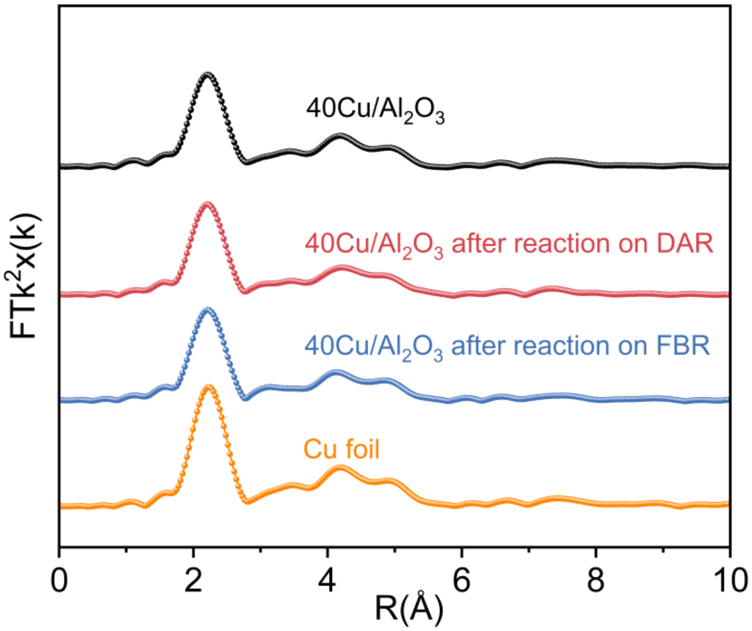

Supplementary Figure 10. Radial distribution function of Cu obtained by Cu K-edge EXAFS data fitting for different samples. (DAR: dynamic activation reactor; FBR: traditional fixed bed reactor)

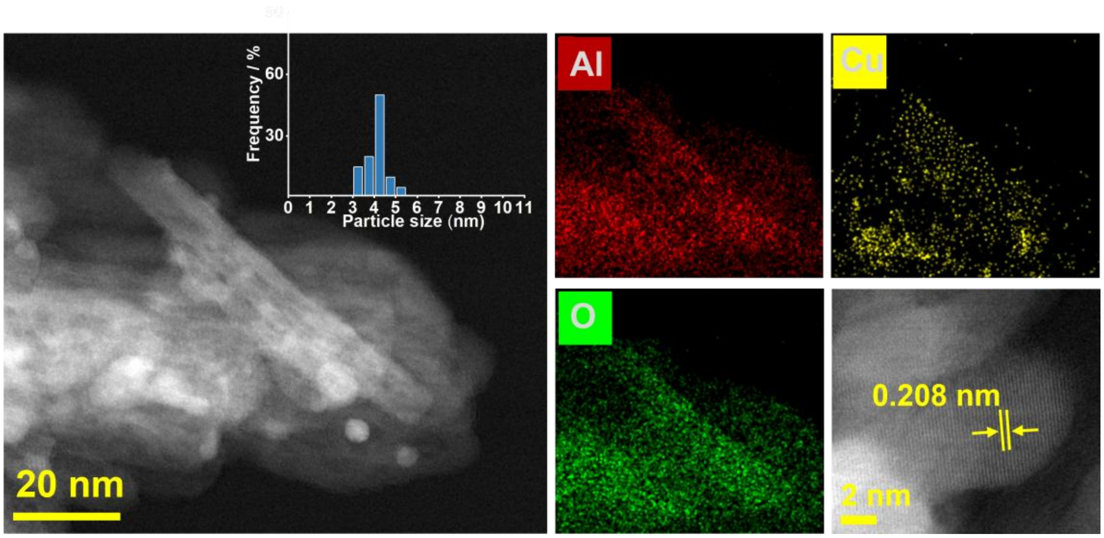

Supplementary Figure 11. Aberration-corrected HADDF-STEM images and elemental mapping of 40Cu/Al_2O_3 after reduction by 10% H_2/N_2 at 300 °C.

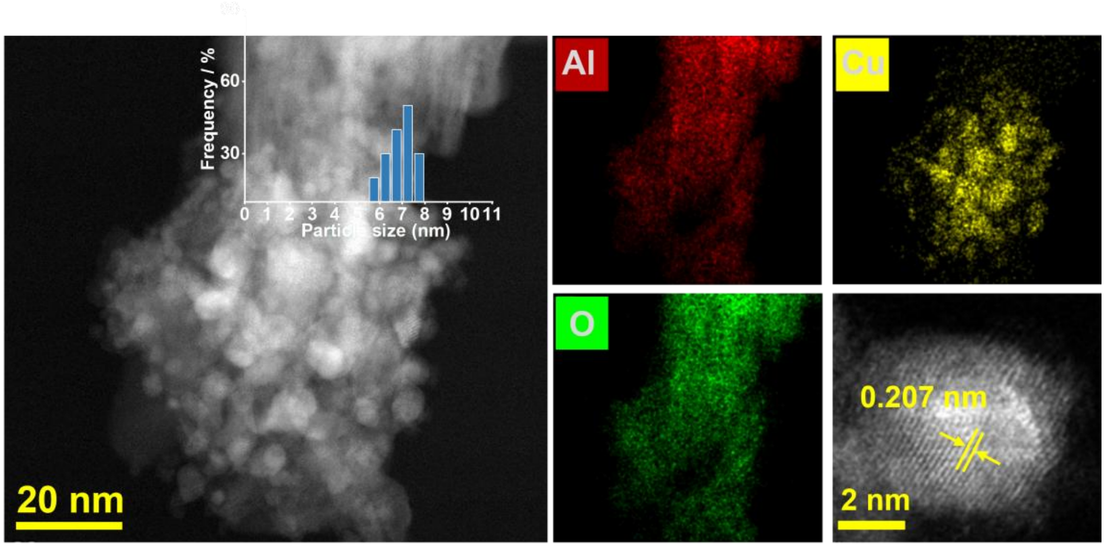

Supplementary Figure 12. Aberration-corrected HADDF-STEM images and elemental mapping of 40Cu/Al_2O_3 after CO_2 hydronation reaction using traditional fixed-bed reactor.

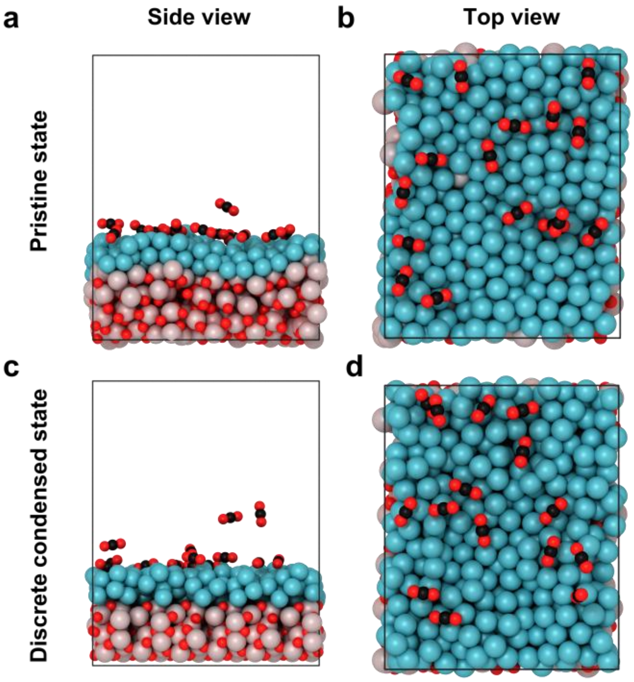

Supplementary Figure 13. Top and side views of snapshots of molecular dynamics simulations on the CO_2 adsorbed on the 40Cu/Al_2O_3 in different states. For the discrete condensed state of Cu, its surface coordination number can be obtained as 7.02. (Red, ivory, black and cyan balls represent O, Al, C and Cu atoms, respectively.)

Supplementary Table 1. BET specific surface area of various samples.

Samples	BET specific surface area (m^2/g)
40Cu/Al_2O_3-fresh	63.4
40Cu/Al_2O_3(DAR)-used	69.8
40Cu/Al_2O_3(FBR)-used	56.3

Supplementary Table 2. XRD refinement data

Samples	Lattice parameters (Å)	Cell volume (Å^3)	Microstress	Bond length (Å)	wrp	χ^2
40Cu/Al_2O_3[a]	3.605	46.87	769.6	2.551	6.216	3.29
40Cu/Al_2O_3 [b]	3.616	47.291	1124.4	2.559	6.155	2.81

[a]The sample was reduced in 10% H_2/N_2 at 300 °C for 3h in fixed bed reactor and the XRD profile was recorded as soon as the sample cooled to room temperature. [b]The XRD profile was recorded as soon as the sample cooled to room temperature, after 2 hours of reaction in dynamic activation reactor (300 °C, 2 MPa, $CO_2/3H_2$, 360 ml/min).

Supplementary Table 3. Cu K-edge EXAFS data of the catalysts [a].

Samples	Shell	R (Å)[b]	CN[c]	σ^2 (Å^2) [d]	ΔE_0 (eV)	r-factor
40Cu-fresh	Cu-Cu	2.535±0.005	9.7±0.7	0.0075	2.27	0.009
40Cu (DAR)-used	Cu-Cu	2.538±0.005	7.8±0.7	0.0085	2.41	0.009
40Cu (FBR)-used	Cu-Cu	2.534±0.006	9.8±0.8	0.0079	2.05	0.011
Cu foil	Cu-Cu	2.537±0.007	12	0.009	5.18	0.012

[a:] The S_0^2 value are 0.869 according to the experimental EXAFS fit of Cu foil by fixing *CN* as the known crystallographic value for Cu K-edge EXAFS spectra fitting, respectively; [b:] bond length; [c:] coordination number; [d:] Debye-Waller factor.

I. Model System and Calculation Details

MD simulations

Molecular dynamics simulations were performed using the Large-scale Atomic/Molecular Massively Parallel Simulator (LAMMPS)[1]. Moltemplate[2] software was used to construct boxes with 25, 30 and 40 Å in the x, y, and z directions, respectively, and the thickness of the vacuum layer in the z direction was set to 25 Å to avoid the periodic influence. The EAM potential[3] was employed to describe the interactions between Al_2O_3 atoms and between Cu-Cu atoms, and the OPLS-AA force field was used to describe the interaction between carbon monoxide and hydrogen molecules. The atomic charge distribution of the atomic force field of carbon monoxide and hydrogen was calculated online using the LigParGen[4,5] website based on the 1.14*CM1A-LBCC method. Visualization of results using OVITO[6] software. The Lennard-Jones (LJ6-12) potential is used to calculate the nonbonding interactions between Cu, Al_2O_3, and gas molecules with a cut-off radius of 12 Å. The spatial summation of the electrostatic potential is based on the Ewald method. In order to correctly reflect the dynamic changes of Cu atoms on the surface during the impact process, an upward force was set to qualitatively describe the force process of Cu hitting the surface of Al_2O_3 during the kinetic relaxation process according to the results of fluid dynamics simulation. Since the MD simulation is mainly to obtain the metastable state at the moment of collision to analyze the influence of this impact process on the surface properties of the catalyst, we assume that the collision is an elastic collision, and the magnitude of the force is calculated by the formula $F=\frac{2v}{\Delta t}$ (Eq. 1), v is the velocity at the moment of collision, and t is the timestep of 1fs which is the smallest unit in the MD simulation. The whole simulation was carried out under the NPT ensemble, the system pressure was set to 2 MPa, and the total simulation time was 1 ns.

Fluid dynamics simulation calculations

The fluid dynamics simulations were performed by Fluent software. The impact simulation of the particles was performed by the discrete phase model method and the viscosity model was performed by the k-epsilon model. According to the results of particle size analysis, the average particle size of the catalyst was 100 μm, so rosin-Rammler method was used to randomly generate nanoparticles with particle sizes of 50 μm and 200 μm with the average size of 100 μm. The catalyst particles were generated from the inlet, with the pressure and temperature at the reactor outlet maintained at 2 MPa and 300°C, respectively. The gas flow rate at the inlet was simulated to

achieve a velocity of 452 m/s, which drove the catalyst particles to accelerate toward the target for collision. Meanwhile, the coupling calculation of pressure and velocity was solved by second-order coupling method.

In the Computational Methods section, we have supplemented detailed simulation settings as follows: The simulation employed double-precision calculations, utilizing hexahedral mesh generated via the ICEM module of Ansys 2022R1, comprising 560,000 cells, with refined mesh density around the target to enhance resolution. The impact process was simulated using the FLUENT module, with catalyst particles modeled via the Discrete Phase Model (DPM). Spatial discretization was performed using the Green-Gauss cell-based method, and flux calculations adopted the distance-based scheme proposed by Rhie-Chow.

Imagine the catalyst particles as copper loaded alumina with a diameter of 20 nanometers and a length of 100 nanometers, with a density of 4 g/cm^3. In 40wt.% Cu content, a particle contains ~13000 Cu atoms. The kinetic energy of one particle in 75 m/s velocity is: $E_{Cu} = \frac{1}{2}\rho\pi r^2 L v^2$ (Eq. 2), where E_k is the kinetic energy of a particle, measured in Joule, ρ is the density, r and L is respectively the radius and length of the catalyst nanorod, and v is the particle velocity, in meters per second (m/s). The E is estimated as ~$3.5*10^{-16}$ J, i.e., ~1840 eV. If the particle is totally stopped at the collision and a half kinetic energy is absorbed by the particle and ~one over three copper atoms is affected by the collision, the average energy felt by each copper atom is about 0.25 eV. The actual energy should be less than this value. Generally, we concluded that the energy of collision was enough to change the surface structure of copper, but not enough to increase the locale temperature too much, upon the collision.

To further validate this, we employed density functional theory (DFT) to compare the energy difference between the discrete condensed state and the normal Cu structure. The discrete condensed Cu structure was extracted from MD simulation snapshots and fully optimized via DFT (K-POINTS = 5×5×1, ENCUT = 800 eV). To isolate the energy change of Cu, the support (Al_2O_3) was removed, and the system energy was calculated at higher precisions. The results show that the energy of the discrete condensed Cu is 0.17 eV/atom higher than that of the normal Cu structure, closely matching the estimated input energy (0.25 eV/atom). This indicates that, theoretically, the collision energy in DAR is sufficient to induce a transition of the Cu surface structure to the discrete condensed state.

Additionally, we simulated Cu layers (2–4 atomic layers) supported on Al_2O_3 to calculate the energy required to remove a single Cu atom, using the following formula :$E_{vac}=E_{defect}+E_{iso}-E_{perfect}$ (Eq. 3), where E_{vac} is the energy required to completely remove a Cu atom (eV), E_{defect} is the energy after removing a Cu atom from the surface of the complete structure (eV), E_{iso} is the the energy of a single Cu atom in a vacuum (eV), and $E_{perfect}$ is the energy of the complete structure (eV).

The calculated vacancy formation energies at the 2nd, 3rd, and 4th Cu layers are 5.27 eV, 5.07 eV, and 5.79 eV, respectively. These energies far exceed the collision energy in DAR, suggesting that mechanical impacts or milling (e.g., in SBMR) are unlikely to completely dislodge Cu atoms from the lattice but are sufficient to elongate Cu-Cu bond lengths or alter the crystalline structure. To explore this, we constructed a model with three Cu layers (60 Cu atoms) on Al_2O_3, generating an amorphous Cu structure via rapid annealing at 300–2500 K, followed by DFT global optimization. Compared to the traditionally supported Cu structure, the amorphous Cu structure exhibits an energy increase of 2.76 eV, which exceeds the input energy of DAR (~0.34 W) but is achievable in the stirred ball mill reactor (SBMR, ~90 W motor power). This indicates that the excessive energy in SBMR disrupts the Cu crystalline structure entirely, whereas the mild collisions in DAR precisely modulate Cu into the discrete condensed state, preserving catalytic activity.

Computational methods

All DFT calculations were performed through the *Vienna Ab initio Simulation Package* (VASP 5.4.4)[7,8,] employing the generalized gradient approximation (GGA) with Perdew-Burke-Ernzerhoff (PBE) exchange-correlation functional[9]. Valence electrons were described by the plane-wave basis sets with the kinetic cut-off energy of 400 eV, while core electrons were represented by the projector augmented-wave pseudopotentials (PAW). A Monkhorst-Pack (3 × 3 × 1) Γ-centered *k*-points grid was used for the integration of Brillouin zone. The adsorption reaction energies (ΔE) and the activation barriers (Ea) were defined as the energy differences between the initial states (ISs) and final states (FSs), and the energy differences between the initial states and transition states (TSs), respectively. The transition states were searched by the climbing-image nudged elastic band (CI-NEB) method[10] together with the improved dimer method[11] and finally verified by the frequency analysis.

Computational models

The alumina support was modelled by a slab of the (111) termination of γ-Al_2O_3 with three layers, and the vacuum layer was set 20 Å. The atoms in the two bottom layers were frozen in their bulk positions during the structural optimization, while the rest atoms were fully relaxed. A supported model with a Cu_{38} cluster of spheroid shape depositing on the surface of γ-Al_2O_3 was adopted for the modelling of Cu/Al_2O_3 in normal traditional fixed bed reactor, which was labeled as Cu/Al_2O_3-pristine. As for the modelling of Cu/Al_2O_3 in the dynamic activation reactor, one frozen frame of Cu/Al_2O_3 during the dynamic simulation was selected and marked as Cu/Al_2O_3-percussive.

II. Supplementary References

1. Thompson, A.P., et al., LAMMPS - a flexible simulation tool for particle-based materials modeling at the atomic, meso, and continuum scales. *Comput. Phys. Commun*. **271**, (2022).
2. Jewett, A.I., et al., Moltemplate: A tool for coarse-grained modeling of complex biological matter and soft condensed matter physics. *J. Mol. Biol*. **433**, (2021).
3. Dodda, L.S., et al., LigParGen web server: an automatic OPLS-AA parameter generator for organic ligands. *Nucleic Acids Res.* **45**, W331-W336 (2017).
4. Etesami, S.A. and E. Asadi, Molecular dynamics for near melting temperatures simulations of metals using modified embedded-atom method. J. Phys. *Chem. Solids.* **112**, 61-72 (2018).
5. Dodda, L.S., et al., 1.14*CM1A-LBCC: Localized bond-charge corrected CM1A charges for condensed-phase simulations. *J. Phys. Chem. Biophys*. **121**, 3864-3870 (2017).
6. Stukowski, A., Visualization and analysis of atomistic simulation data with OVITO–the Open Visualization Tool. *Modell. Simul. Mater. Sci. Eng*. **18,** 015012 (2010).
7. Kresse, G. and J. Furthmüller, Efficient iterative schemes for ab initio total-energy calculations using a plane-wave basis set. *Phys. Rev. B*. **54**, 11169-11186 (1996).
8. Kresse, G. and J. Furthmüller, Efficiency of ab-initio total energy calculations for metals and semiconductors using a plane-wave basis set. *Comput. Mater. Sci.* **6**, 15-50 (1996).
9. Perdew, J.P., K. Burke, and M. Ernzerhof, Generalized gradient approximation made simple. Phys. *Rev. Lett.* **77**, 3865-3868 (1996).
10. Henkelman, G. and H. Jónsson, Improved tangent estimate in the nudged elastic band method for finding minimum energy paths and saddle points. *J. Chem. Phys.* **113**, 9978-9985 (2000).
11. Henkelman, G. and H. Jonsson, A dimer method for finding saddle points on high dimensional potential surfaces using only first derivatives. *J. Chem. Phys.* **111**, 7010-7022 (1999).
